# Atrial Myopathy and Heart Failure: Immunomolecular Mechanisms and Clinical Implications

**DOI:** 10.3390/ijms26178210

**Published:** 2025-08-24

**Authors:** Marta Gil Fernández, Andrea Bueno Sen, Paula Cantolla Pablo, Almudena Val Blasco, Gema Ruiz Hurtado, Carmen Delgado, Carolina Cubillos, Lisardo Boscá, María Fernández Velasco

**Affiliations:** 1 Clinical and Invasive Cardiology Research Group (ICCI-PAZ), Hospital La Paz Institute for Health Research (IdiPAZ), 28046 Madrid, Spain; andreabueno_24@hotmail.com (A.B.S.); cantolla.paula@gmail.com (P.C.P.); almudena.valblasco@gmail.com (A.V.B.); 2 Cardiovascular Biomedical Research Centre Network (CIBERCV), Instituto de Salud Carlos III (ISCIII), 28029 Madrid, Spain; cdelgado@iib.uam.es (C.D.); lbosca@iib.uam.es (L.B.); 3 Cardiorenal Translational Laboratory, Research Institute Hospital 12 de Octubre (i+12), 28041 Madrid, Spain; gemaruiz@h12o.es; 4 Sols-Morreale Biomedical Research Institute (IIBM), Consejo Superior de Investigaciones Científicas (CSIC)-Universidad Autónoma de Madrid (UAM), 28029 Madrid, Spain; 5 Respiratory Diseases Group, Respiratory Diseases Department, Hospital La Paz Institute for Health Research (IdiPAZ), 28046 Madrid, Spain; cubilloszapata@gmail.com; 6 Biomedical Research Networking Center for Respiratory Diseases (CIBERES), Instituto de Salud Carlos III (ISCIII), 28029 Madrid, Spain

**Keywords:** atrial myopathy, heart failure, inflammation, innate immunity

## Abstract

Heart failure (HF) remains a major global health challenge defined by the inability of the heart to adequately meet systemic metabolic requirements. While ventricular dysfunction has traditionally been the primary focus in both conceptual and clinical frameworks of HF, emerging evidence highlights atrial myopathy—covering structural, functional, electrical, metabolic, and neurohormonal remodeling—as a central yet often overlooked contributor to disease progression across the HF spectrum. This review offers a comprehensive overview of the molecular and cellular mechanisms underlying atrial remodeling, with a focus on inflammation and innate immune activation as key pathogenic mediators. Among pattern recognition receptors, Toll-like receptors (TLRs) and NOD-like receptors (NLRs) play crucial roles in translating myocardial stress into pro-inflammatory, profibrotic, and pro-arrhythmic signals that exacerbate HF. By combining experimental and clinical evidence, we emphasize atrial myopathy as both a biomarker and an active driver of HF deterioration, advocating for the inclusion of atrial-targeted diagnostics and immunomodulatory therapies in future HF treatment approaches. Such a paradigm shift holds significant potential for improved risk stratification, arrhythmia prevention, attenuation of structural remodeling, and ultimately, better prognosis and clinical outcomes in this increasingly common syndrome.

## 1. Introduction

Heart failure (HF) is a complex clinical syndrome defined by impaired cardiac output leading to inadequate peripheral perfusion, resulting in increasing morbidity and mortality worldwide [1,2]. Despite advances in treatment, outcomes remain inadequate, highlighting the need for deeper mechanistic understanding. While traditionally linked to ventricular dysfunction, growing evidence points to atrial myopathy—characterized by structural, functional, electrical, metabolic, and neurohormonal abnormalities—as a key factor in HF pathophysiology. Notably, atrial remodeling not only reflects disease severity but also actively promotes HF progression, independent of left ventricular (LV) function [3,4,5]. Recent phenotyping studies have revealed that atrial myopathy is present in a significant subset of patients with chronic HF. In the ELMSTAT-HF study, which analyzed over 2300 patients, approximately 15% were classified within a distinct phenotype characterized by atrial myopathy as the dominant feature [6]. This review explores emerging knowledge about the relationship between atrial remodeling and innate immune activation, focusing on the roles of Toll-like receptors (TLRs) and NOD-like receptors (NLRs) in mediating pro-inflammatory and fibrotic responses as well as their role in atrial fibrillation (AF). We suggest a paradigm shift that considers atrial myopathy a crucial target for diagnosis and therapy in HF.

## 2. Definition, Classification, and Clinical Overview of Heart Failure

HF is a progressive syndrome caused by structural and/or functional myocardial impairment that ultimately leads to inadequate systemic perfusion [1]. Affecting over 26 million people worldwide, its prevalence is increasing with aging populations and rising cardiovascular risk factors [2]. HF is the leading cause of hospitalization in individuals over 65 and is associated with high mortality—nearly 50% within five years of diagnosis [7,8].

The syndrome results from a complex interplay of hemodynamic overload, neurohormonal activation, maladaptive remodeling, abnormal calcium handling, extracellular matrix dysregulation, apoptosis, genetic predisposition, and immune dysfunction [9,10]. Classification mainly relies on LV ejection fraction (LVEF): HF with reduced EF (HFrEF; LVEF < 40%), HF with mildly reduced EF (HFmrEF; LVEF 40–49%), and HF with preserved EF (HFpEF; LVEF ≥ 50%) [11,12,13,14,15,16]. While HFrEF is marked by impaired systolic function and eccentric hypertrophy, HFpEF is linked to diastolic dysfunction and concentric remodeling [9]. HFmrEF shows intermediate features and is an area of ongoing research, with emerging evidence indicating partial overlap with both HFpEF and HFrEF phenotypes [17].

## 3. Atrial Remodeling in the Pathophysiology of Heart Failure

The pathogenesis of HF involves various structural, molecular, and cellular changes that impair cardiac function. Although ventricular dysfunction has been the main factor in driving HF progression, increasing evidence emphasizes the critical role of atrial remodeling in disease development and prognosis. The left atrium (LA) acts as a key regulator of LV filling through three distinct phases [18,19]: the reservoir phase, where the LA stores pulmonary venous return during ventricular systole; the conduit phase, characterized by passive flow from the pulmonary veins to the LV in early diastole; and the contractile phase, involving active atrial contraction that enhances ventricular filling in late diastole.

**Figure 1:**
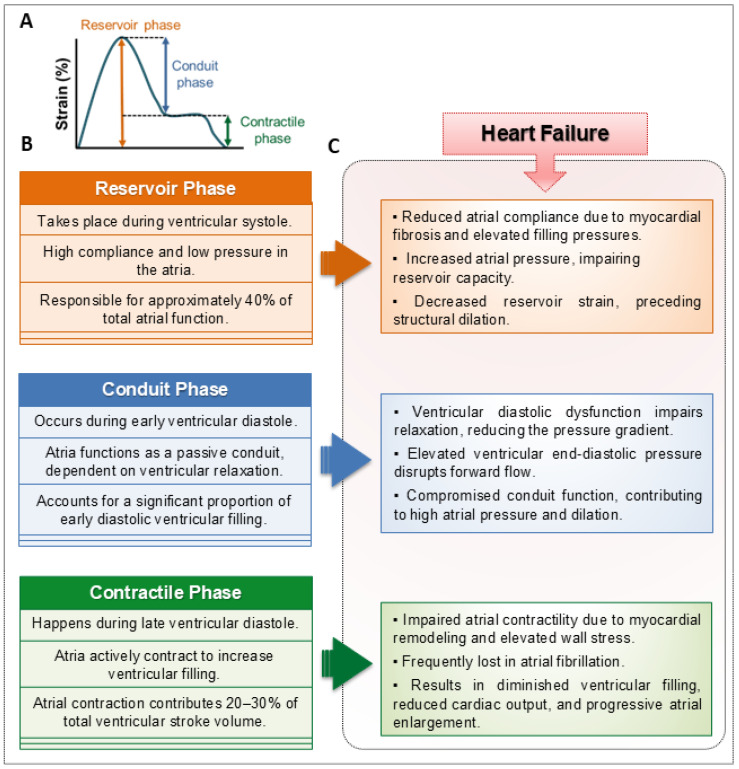
Phasic atrial function and its impairment in heart failure. Panel (**A**) depicts a longitudinal strain curve derived from speckle-tracking echocardiography of the left atrium. The graph shows the three phasic components of atrial function—reservoir, conduit, and contractile. Panel (**B**) schematically illustrates the physiological basis of phasic atrial function. The atria act as a reservoir during ventricular systole, receiving venous return while the atrioventricular valves are closed; as a conduit during early diastole, allowing passive blood transfer into the ventricles; and as a contractile chamber during late diastole, enhancing ventricular preload through active contraction. Panel (**C**) summarizes the progressive impairment of these functions in heart failure (HF). Reservoir strain decreases due to increased atrial stiffness and fibrosis, compounded by elevated ventricular filling pressures. Conduit function deteriorates with worsening ventricular relaxation, leading to atrial pressure overload and chamber dilation. Contractile function is often compromised or absent—especially during atrial fibrillation (AF)—resulting in impaired late diastolic filling and further structural remodeling. Collectively, these changes reflect the integrated pathophysiology of atrial myopathy in HF.

Impairment in any of these stages is common in HF and is linked to increased atrial stiffness, decreased compliance, and higher filling pressures (Figure 1), all of which have negative prognostic implications [20,21]. LA and right atrial (RA) enlargement, often caused by chronic pressure or volume overload, are not only markers of disease severity but also independent predictors of adverse cardiovascular outcomes [22].

Mechanical stretch from elevated atrial pressure triggers maladaptive molecular, cellular, and neurohormonal responses, leading to atrial fibrosis, electrical instability, and inflammation. These processes result in atrial myopathy, worsening the HF phenotype [23]. According to EHRA/HRS/APHRS/SOLAECE expert consensus, atrial myopathy is defined as ‘any complex of structural, architectural, contractile, or electrophysiological changes affecting the atria with the potential to produce clinically relevant manifestations [23]. In the setting of HF, atrial myopathy involves interconnected structural, functional, electrical, metabolic, and neurohormonal disturbances that converge to produce sustained atrial dysfunction [24].

### 3.1. Structural Remodeling of the Atria

Structural remodeling is a key aspect of atrial myopathy, involving architectural changes in response to chronic hemodynamic stressors, mainly pressure and volume overload [25]. Elevated atrial filling pressures cause atrial dilation and hypertrophy, resulting in progressive enlargement [26]. The fibrotic remodeling process is driven by interactions among cardiomyocytes, fibroblasts, endothelial cells, immune cells, and adipocytes, that collectively coordinate the fibrotic reprogramming of atrial tissue [27].

At the cellular level, cardiomyocytes undergo significant changes, including sarcomere disassembly, glycogen buildup, dedifferentiation, and re-expression of fetal gene programs. These alterations impair atrial contractile function and promote mechanical dysfunction. At the same time, fibroblast activation leads to excessive extracellular matrix (ECM) deposition, creating a profibrotic environment that reduces atrial compliance and electrical conductivity [28]. Endothelial cells undergo hypertrophic remodeling, develop gaps between cells, along with increased expression of adhesion molecules that encourage local inflammation and immune cell infiltration [29]. Expansion of adipose tissue and deposition of non-collagenous substances (e.g., amyloid, glycosphingolipids) further raise tissue stiffness and dysfunction [30]. Additionally, granulomatous inflammation may also play a role in structural damage [23].

Chronic inflammation, especially innate immune activation, plays a key role in driving these structural changes. Ongoing inflammatory signals lead to myocyte apoptosis, ECM remodeling, and atrial enlargement, which support the self-perpetuating cycle of atrial myopathy in HF.

### 3.2. Functional Remodeling of the Atria

Functional remodeling involves mechanical and contractile changes that impair atrial function, affecting all three phases: reservoir, conduit, and contractile. In atrial myopathy, these remodeling features include decreased contractility, reduced compliance, mechanical dyssynchrony, and loss of contractile reserve. These issues directly hinder ventricular filling, increase atrial and pulmonary pressures, and worsen HF symptoms.

This remodeling phenotype is closely linked with structural and electrical changes. Fibrosis, cardiomyocyte apoptosis, and dedifferentiation weaken atrial myocardium integrity, resulting in reduced force generation and asynchronous atrial contraction. At the molecular level, functional decline is caused by altered intracellular calcium dynamics, oxidative stress, mitochondrial dysfunction, and impaired excitation–contraction coupling. These changes decrease myocardial efficiency and energy production, leading to hemodynamic instability [31].

Additionally, progressive contractile dysfunction facilitates the development of atrial arrhythmias, especially AF, which further drives atrial remodeling and HF progression [32]. Therefore, functional remodeling is both a result of and a contributor to the pathophysiology of atrial myopathy.

### 3.3. Electrical Remodeling of the Atria

Electrical remodeling in atrial myopathy involves a range of electrophysiological alterations within atrial tissue that promote arrhythmias, especially AF [33,34]. In the context of HF and atrial myopathy, these maladaptive changes develop in response to mechanical stress, inflammation, and neurohormonal activation, together creating a substrate that facilitates the initiation and maintenance of AF [32].

A key hallmark of electrical remodeling is the shortening of the atrial effective refractory period (AERP), which reduces excitation wavelength and facilitates reentrant mechanisms critical for AF [35]. Simultaneously, atrial conduction velocity decreases due to impaired sodium current (I_Na_) and altered gap junction integrity, promoting conduction block and increasing the likelihood of reentrant arrhythmias [32]. Additionally, increased dispersion of repolarization, reflected by heightened heterogeneity in repolarization timing, further predisposes the atria to wave breaks and fibrillatory conduction [32].

Ion channel remodeling involves reductions in L-type calcium current (I_Ca,L_) and increases in inward rectifier potassium current (I_K1_), which impair action potential duration (APD) and raise the risk of early afterdepolarizations and triggered activity [36]. Abnormal calcium handling, marked by excessive sarcoplasmic reticulum leak and ryanodine receptor (RyR2) dysfunction, accelerates structural deterioration and increases susceptibility to delayed afterdepolarizations [37,38]. Gap junction remodeling, thorough downregulation and lateral redistribution of connexin 40 (Cx40)—the main atrial connexin—disrupts anisotropic conduction and induces conduction heterogeneity, promoting arrhythmogenesis [39].

Importantly, imbalances in calcium, potassium, and iron synergistically influence atrial electrophysiology and contribute to HF progression [40]. Altered ion levels can directly or indirectly modulate ion channel activity and expression, potentially worsening electrical instability. Iron metabolism disturbances play a dual role: iron deficiency reduces mitochondrial oxidative capacity and ATP availability, impairing ion pump function such as Na^+^/K^+^-ATPase and calcium cycling, thereby slowing conduction velocity and prolonging APD [41,42,43]. Conversely, iron overload increases reactive oxygen species (ROS) production, leading to oxidative modifications of ion channels and membrane proteins, disrupting membrane excitability and promoting arrhythmogenesis [44,45,46]. Moreover, iron-induced ROS can alter ion channel gene expression and post-translational modifications, intensifying electrical heterogeneity and instability [44,47,48,49].

Inflammatory mediators, including pro-inflammatory cytokines, further worsen electrical remodeling by modulating ion channel expression and function, increasing arrhythmogenic potential [50,51].

### 3.4. Metabolic Remodeling of the Atria

Metabolic remodeling in atrial myopathy reflects a fundamental change in energy substrate use and mitochondrial function, driven by the pathophysiological environment of HF. A key feature of this process is the reversion to a fetal-like metabolic phenotype, characterized by decreased oxidative phosphorylation and a compensatory increase in glycolysis [52,53]. Although initially adaptive, persistent metabolic inefficiency ultimately contributes to atrial stress and dysfunction.

The elevated energy demands resulting from contractile dysfunction and electrical instability cause ATP depletion, disrupting essential cellular processes for atrial electrophysiology and contractility. Reduced energy availability hampers ionic homeostasis, impairing calcium cycling, sodium-potassium pump function, and ion channel activity [54,55]. Mitochondrial dysfunction worsens these effects through oxidative stress, increased ROS production, and mitochondrial permeability transition pore opening, all of which intensify myocyte injury, apoptosis, and profibrotic signaling [56].

Iron homeostasis disturbances are integral to this metabolic remodeling, as noted above. Both iron deficiency and overload adversely affect mitochondrial function and energy metabolism, triggering a cascade of ionic imbalances and electrophysiological disturbances that amplify atrial dysfunction [41,42,43,44,45,46,57]. ROS-mediated activation of signaling pathways, including AMP-activated protein kinase (AMPK) and nuclear factor kappa B (NF-κB), which modulate ion channel gene expression and promote pro-inflammatory and profibrotic responses [44,47,48,49,58].

Lipid metabolic dysregulation, secondary to impaired mitochondrial β-oxidation, results in intracellular accumulation of triglycerides and toxic intermediates such as ceramides and diacylglycerols [59]. These lipids worsen oxidative damage and trigger inflammatory and apoptotic signaling pathways, further compromising mitochondrial function and creating a deleterious metabolic–structural feedback cycle.

Beyond soluble mediators, resident and infiltrating immune cells perpetuate metabolic remodeling by impairing mitochondrial function and oxidative phosphorylation, reducing ATP production and undermining cardiomyocyte viability and contractility [60,61,62]. Macrophage-derived cytokines, including tumor necrosis factor-alpha (TNF-α) and interleukin-6 (IL-6), impair mitochondrial oxidative phosphorylation and promote a glycolytic shift, resulting in energetic inefficiency, oxidative stress, and apoptosis [60,63]. In parallel, infiltrating T lymphocytes and neutrophils promote fibroblast activation, ECM deposition, and ROS-mediated mitochondrial damage [64,65,66,67]. This chronic inflammatory-metabolic crosstalk creates a hostile microenvironment that further worsens structural, electrical, and functional deterioration in HF-associated atrial myopathy.

Together, these metabolic alterations compromise atrial contractility, promote electrical instability, and perpetuate the self-reinforcing cycle of atrial myopathy. Metabolic remodeling thus represents a critical contributor to the pathogenesis of atrial dysfunction in HF, integrating mitochondrial impairment, energy imbalance, oxidative damage, disrupted ion homeostasis, lipotoxicity and inflammation.

### 3.5. Neurohormonal Remodeling of the Atria

Neurohormonal dysregulation is a key factor driving atrial remodeling in HF, cleading to structural, electrical, functional, and metabolic changes. The failing atrium faces a complex neurohormonal environment, including increased levels of atrial natriuretic peptide (ANP), brain natriuretic peptide (BNP), angiotensin II (Ang-II), aldosterone, transforming growth factor-beta (TGF-β), and heightened sympathetic nervous system activity [25].

ANP and BNP are released in response to atrial stretch and initially provide protective effects by promoting vasodilation, natriuresis, and antifibrotic signaling. However, persistently elevated BNP levels—commonly observed in HF—are associated with LA enlargement, impaired contractility, and worse prognosis [68,69]. Chronic elevation may indicate a maladaptive compensatory response linked to progressive atrial pathology.

In contrast, the renin–angiotensin–aldosterone system (RAAS) plays a directly pathogenic role in atrial myopathy. Ang-II and aldosterone promote fibroblast proliferation, collagen synthesis, and ECM deposition, thereby worsening atrial fibrosis and structural remodeling. TGF-β, a downstream effector of RAAS activation, is a key mediator of profibrotic and pro-inflammatory signaling, contributing to myocyte hypertrophy, endothelial dysfunction, and increased thromboembolic risk [70,71].

Sympathetic overactivation is another key feature of HF-related neurohormonal imbalance. Chronic adrenergic stimulation increases myocardial oxygen demand while reduing β-adrenergic receptor responsiveness, further disturbing excitation–contraction coupling and promoting electrical instability. Elevated catecholamines worsen calcium mishandling, boosting triggered activity and arrhythmogenesis [72]. Importantly, this sympathetic dominance is accompanied by parasympathetic withdrawal, which disrupts a key regulatory mechanism of atrial electrophysiology. Reduced vagal tone decreases heart rate variability, shortens atrial refractory periods, and promotes reentrant circuits—all of which support the initiation and maintenance of AF. The loss of cholinergic modulation also impairs anti-inflammatory signaling and baroreflex sensitivity, thereby worsening autonomic imbalance and increasing atrial vulnerability.

Additionally, sustained neurohormonal activation disrupts metabolic homeostasis, exacerbating mitochondrial dysfunction, increasing oxidative stress, and fueling the energetic imbalance characteristic of atrial myopathy. Crosstalk between neurohormonal and inflammatory pathways further amplifies maladaptive remodeling, creating a self-perpetuating cycle of atrial dysfunction.

Neurohormonal remodeling thus encompasses multiple maladaptive pathways—fibrosis, inflammation, autonomic imbalance, calcium dysregulation, and energetic failure—that collectively drive the progression of atrial myopathy in HF.

## 4. Innate Immune Response and Inflammation in Heart Failure-Associated Atrial Myopathy

**Figure 2:**
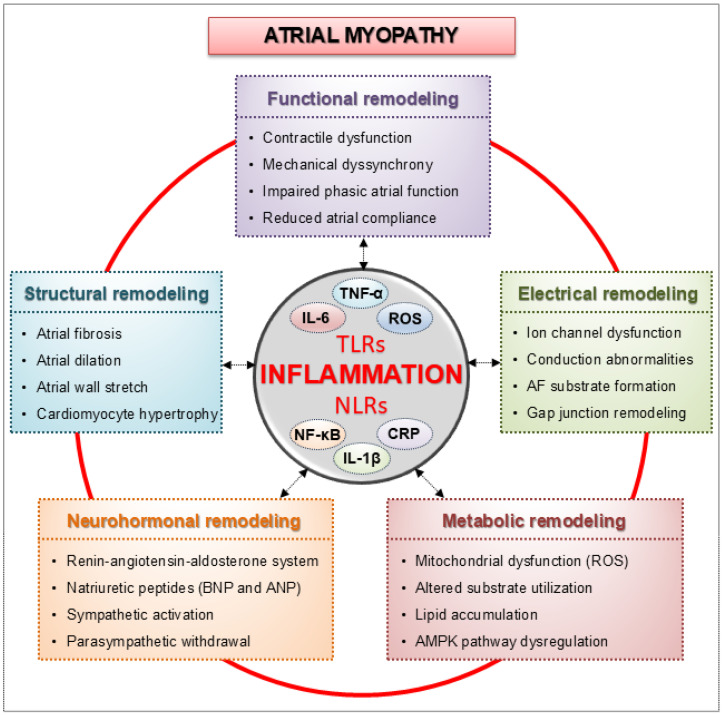
Inflammation as a central driver of atrial remodeling in atrial myopathy. The schematic shows the two-way interaction between inflammation and the five main domains of atrial remodeling: structural, electrical, functional, metabolic, and neurohormonal. Innate immune activation via Toll-like receptors (TLRs) and NOD-like receptors (NLRs) triggers cytokine release [interleukin-1β (IL-1β), interleukin-6 (IL-6), tumor necrosis factor-α (TNF-α)], reactive oxygen species (ROS) generation, nuclear factor κB (NF-κB) signaling, and C-reactive protein (CRP) elevation, thereby driving fibrosis, ion channel and gap junction dysfunction, mitochondrial impairment, and neurohormonal activation. These maladaptive processes culminate in atrial myopathy, while a feed-forward loop sustains inflammation and remodeling, promoting atrial dysfunction and arrhythmogenesis in heart failure (HF).

These remodeling processes—structural, functional, electrical, metabolic, and neurohormonal—are tightly interconnected, creating a complex pathological network that drives the progression of atrial myopathy. At the core of this dynamic interplay is inflammation, which acts both as an initiator and an amplifier of maladaptive remodeling in the failing atrium (Figure 2). The inflammatory response is triggered by various cellular stressors, including mechanical stretch, oxidative damage, mitochondrial dysfunction, and neurohormonal activation. In recent years, increasing evidence has emphasized the role of the innate immune system in cardiovascular disease, especially in the context of HF, given its close association with heightened inflammatory responses [73,74].

### 4.1. Pattern Recognition Receptors: Innate Immune Sensors in Atrial Remodeling

The innate immune system includes pattern recognition receptors (PRRs) that detect tissue injury and trigger the inflammatory response. These receptors identify conserved molecular motifs, including pathogen-associated molecular patterns (PAMPs) and damage-associated molecular patterns (DAMPs) released during sterile injury [75]. Five main PRR families have been identified based on their structural domains: TLRs, C-type lectin receptors (CLRs), NLRs, absent in melanoma 2 (AIM2)-like receptors, and retinoic acid-inducible gene-I (RIG-I)-like receptors [76].

Membrane-bound TLRs and CLRs monitor extracellular and endosomal compartments, while cytosolic AIM2-like receptors, RIG-I-like receptors, and NLRs serve as intracellular sentinels. When activated, these receptors trigger intracellular signaling pathways—primarily through NF-κB and mitogen-activated protein kinase (MAPK) pathways—which lead to the production of pro-inflammatory cytokines and modulate cellular stress responses. Under pathological conditions, this persistent signaling fosters chronic inflammation and maladaptive cardiac remodeling.

### 4.2. Toll-like and NOD-like Receptors in the Failing Heart

Among PRRs, TLRs and NLRs have emerged as key regulators of myocardial inflammation and remodeling in cardiovascular disease, including HF [73,74]. Chronic TLR activation has been linked to cardiac dysfunction, partly through modulation of ion channel activity and the promotion of adverse electrical remodeling. For instance, TLR-2 stimulation induces cardiac inflammation and contractile impairment in vitro [77], while TLR-4 activation prolongs APD by primarily downregulating the transient outward potassium current (I_to_) and enhancing calcium efflux through the sodium/calcium exchanger (NCX). These changes promote arrhythmogenic events such as delayed afterdepolarizations and triggered activity [78]. Both pharmacological inhibition and genetic silencing of TLR-4 have been shown to reverse these abnormalities, improving cardiac function and reducing arrhythmia susceptibility [79,80]. Elevated TLR-2 expression has also been documented in RA tissue from patients with persistent and paroxysmal AF undergoing valve surgery [81], further implicating TLR signaling in atrial pathology.

Beyond TLRs, the role of NLRs—particularly the NLRP3 inflammasome—in cardiac pathology is increasingly recognized. The NLRP3 inflammasome is a cytosolic multiprotein complex that activates caspase-1 (CASP1), leading to the maturation and release of pro-inflammatory cytokines interleukin-1β (IL-1β), and interleukin-18 (IL-18). NLRP3 activation has been linked to atrial fibrosis, dilation, and a higher AF burden in both humans and experimental models [82,83]. Notably, the functional consequences of NLRP3 activation are cell-type specific: in cardiomyocytes, it impairs contractile function and relaxation while promoting arrhythmogenic remodeling, whereas in immune cells, NLRP3 regulates host defense by modulating essential immune processes, including cell migration and efferocytosis [84,85].

A compelling study using cardiomyocyte-specific knock-in mice overexpressing a constitutively active form of NLRP3 (*Myh6:Nlrp3*^A350V/+^) demonstrated spontaneous development of atrial hypertrophy, fibrosis, and increased susceptibility to AF. This phenotype was associated with upregulation of CASP1 and enhanced arrhythmogenicity during electrical stimulation. Importantly, genetic ablation of NLRP3 reversed these pathological features, confirming its key role in the development of atrial myopathy [85].

### 4.3. Inflammatory and Pro-Resolving Mediators Involved in Heart Failure and Atrial Myopathy

Activation of PRRs triggers downstream signaling pathways that lead to the production of pro-inflammatory cytokines such as TNF-α, IL-6, IL-1β, and TGF-β [27,86]. These cytokines stimulate fibroblast activation, myofibroblast differentiation, and ECM deposition, thereby promoting atrial fibrosis, disrupting atrial structure, and impairing mechanical function.

Under physiological conditions, inflammation progresses through a coordinated sequence of acute, resolution, and reparative phases. However, disruption of this tightly regulated process—particularly a failure to resolve the acute phase—leads to chronic inflammation, a key driver of HF progression [87]. Ongoing activation of innate immunity sustains the release of inflammatory cytokines including IL-1β, IL-6, IL-18, and TNF-α, which have hypertrophic, fibrotic, and pro-apoptotic effects on cardiomyocytes [88]. Notably, atrial cardiomyocytes are not passive targets but active participants in the inflammatory response, secreting cytokines and expressing their corresponding receptors (IL-1R1, IL-6R, IL-18R, TNFR), thus creating a self-amplifying inflammatory loop [89,90].

Experimental data support the functional effects of this inflammatory signaling. IL-6 has been shown to induce reversible electrical remodeling in atrial myocytes by downregulating connexin expression [91], while IL-1β and IL-18 contribute to AF pathogenesis by promoting atrial structural and electrical remodeling [90]. TNF-α, extensively studied in HF, promotes atrial dilation, fibrosis, and conduction abnormalities [92]. Transgenic mice with cardiomyocyte-specific TNF-α overexpression exhibit impaired atrial contractility and increased AF susceptibility [93], while TNF-α blockade reduces adverse atrial remodeling in experimental HF models [94].

In recent years, attention has shifted from focusing solely on pro-inflammatory agents in cardiovascular disease to recognizing the vital role of inflammation resolution mechanisms. It is now well established that a failure to effectively resolve inflammation significantly contributes to the development and progression of cardiovascular conditions, including HF and atrial remodeling. Particular interest has emerged in the role of specialized pro-resolving mediators (SPMs)—a subgroup of endogenous lipid-derived molecules such as resolvins, protectins, maresins, and lipoxins—which coordinate the active resolution phase of inflammation without impairing host defense mechanisms [95,96,97,98,99].

Unlike traditional anti-inflammatory therapies that broadly suppress immune responses, SPMs actively coordinate the removal of inflammatory cells, restore tissue balance, and trigger healing pathways, thus preventing chronic inflammation. In HF, unresolved inflammation leads to atrial structural changes, fibrosis, and electrical instability, all of which are key features of atrial myopathy and AF. Notably, Resolvin D1 has been shown to reduce atrial remodeling and lower AF risk after myocardial infarction. Early use of Resolvin D1 provided protection to both atrial and ventricular compartments, while delayed treatment offered benefits mainly to the atria [100]. Similarly, in rheumatic heart disease, Resolvin D1 prevented RA remodeling by decreasing inflammation, fibrosis, and electrical disturbances [100]. Impaired production or signaling of SPMs may worsen inflammation and remodeling in cardiovascular disease, indicating that a failure of resolution pathways is a major factor in disease progression and highlighting these mediators as promising therapeutic targets for HF and atrial remodeling [97].

## 5. Conclusions

Atrial myopathy is a key yet often overlooked element of HF pathophysiology. Besides being linked to ventricular dysfunction, atrial myopathy—including structural, functional, electrical, metabolic, and neurohormonal changes—actively contributes to disease progression, promotes arrhythmia development, and worsens clinical outcomes. In this regard, several clinical trials are currently evaluating immunomodulatory strategies in cardiovascular disease, including IL-6 blockade with ziltivekimab, selective NLRP3 inhibitors such as dapansutrile, and established anti-inflammatory agents such as colchicine and canakinumab [101,102,103].

This review emphasizes the central role of the innate immune system, especially TLRs and NLRs, in driving this harmful remodeling, inflammation, fibrosis, and electrical instability. Recognition of these immunomolecular mechanisms opens promising therapeutic avenues for developing targeted interventions that aim to modulate atrial inflammation and remodeling, reducing arrhythmogenic risk, and ultimately improve clinical outcomes. Moving forward, clinical translation should focus on immunomodulatory strategies, biomarker-guided precision medicine, and routine evaluation of atrial function as key components of HF management. Reframing atrial myopathy as a modifiable, pathogenic driver rather than a secondary consequence of HF could fundamentally shift current paradigms and enable more effective, mechanism-based interventions.

## Data Availability

Not applicable.

## Abbreviations

The following abbreviations are used in this manuscript:

AERP	Atrial effective refractory period
AF	Atrial fibrillation
AIM2	Absent in melanoma 2
AMPK	AMP-activated protein kinase
Ang-II	Angiotensin II
ANP	Atrial natriuretic peptide
APD	Action potential duration
BNP	Brain natriuretic peptide
CASP1	Caspase-1
CLR	C-type lectin receptor
Cx40	Connexin 40
DAMPs	Damage-associated molecular patterns
ECM	Extracellular matrix
HF	Heart failure
HFmrEF	Heart failure with mildly reduced ejection fraction
HFpEF	Heart failure with preserved ejection fraction
HFrEF	Heart failure with reduced ejection fraction
ICa,L	L-type calcium current
IK1	Inward rectifier potassium current
IL-18	Interleukin-18
IL-1β	Interleukin-1 beta
IL-6	Interleukin-6
INa	Sodium current
LA	Left atrium
LV	Left ventricle
LVEF	Left ventricular ejection fraction
MAPK	Mitogen-activated protein kinase
NF-κB	Nuclear factor kappa-B
NLR	NOD-like receptor
PAMPs	Pathogen-associated molecular patterns
PRR	Pattern recognition receptor
RA	Right atrium
RAAS	Renin–angiotensin–aldosterone system
RIG-I	Retinoic acid-inducible gene I
ROS	Reactive oxygen species
RyR2	Ryanodine receptor type 2
TGF-β	Transforming growth factor-beta
TLR	Toll-like receptor
TNF-α	Tumor necrosis factor-alpha
